# Small amount of alcohol did not deteriorate microsurgical dexterity: a prospective laboratory study

**DOI:** 10.1007/s00701-023-05501-0

**Published:** 2023-02-09

**Authors:** Ville Vasankari, Christian Eisenring, Tobias Rossmann, Michael Veldeman, Ville Nurminen, Ahmad Hafez, Rahul Raj, Mika Niemelä, Martin Lehecka

**Affiliations:** 1 Department of Neurosurgery, Helsinki University Hospital and University of Helsinki, P.O. Box 266, 00029 Helsinki, Finland; 2 Department of Neurosurgery, Hirslanden Hospital, Zurich, Switzerland; 3 Department of Neurosurgery, Neuromed Campus, Kepler University Hospital, Johannes Kepler University, Linz, Austria; 4 Department of Neurosurgery, RWTH Aachen University Hospital, Aachen, Germany

**Keywords:** Alcohol, Microsurgery, Microsurgical dexterity, Neurosurgery, Suturing, Tremor

## Abstract

**Background:**

Alcohol consumption has been reported to deteriorate surgical performance both immediately after consumption as well as on the next day. We studied the early effects of alcohol consumption on microsurgical manual dexterity in a laboratory setting.

**Method:**

Six neurosurgeons or neurosurgical residents (all male) performed micro- and macro suturing tasks after consuming variable amounts of alcohol. Each participant drank 0–4 doses of alcohol (14 g ethanol). After a delay of 60–157 min, he performed a macrosurgical and microsurgical task (with a surgical microscope). The tasks consisted of cutting and re-attaching a circular latex flap (diameter: 50 mm macrosuturing, 4 mm microsuturing) with eight interrupted sutures (4–0 multifilament macrosutures, 9–0 monofilament microsutures). We measured the time required to complete the sutures, and the amplitude and the frequency of physiological tremor during the suturing. In addition, we used a four-point ordinal scale to rank the quality of the sutures for each task. Each participant repeated the tasks several times on separate days varying the pre-task alcohol consumption (including one sober task at the end of the data collection).

**Results:**

A total of 93 surgical tasks (47 macrosurgical, 46 microsurgical) were performed. The fastest microsurgical suturing (median 11 min 49 s, [interquartile range (IQR) 654 to 761 s]) was recorded after three doses of alcohol (median blood alcohol level 0.32‰). The slowest microsurgical suturing (median 15 min 19 s, [IQR 666 to 1121 s]) was observed after one dose (median blood alcohol level 0‰). The quality of sutures was the worst (mean 0.70 [standard deviation (SD) 0.48] quality points lost) after three doses of alcohol and the best (mean 0.33 [SD 0.52] quality points lost) after four doses (median blood alcohol level 0.44‰).

**Conclusions:**

Consuming small amount of alcohol did not deteriorate microsurgical performance in our study. An observed reduction in physiological tremor may partially explain this.

**Supplementary Information:**

The online version contains supplementary material available at 10.1007/s00701-023-05501-0.

## Introduction

Microsurgical techniques are an essential part of modern microneurosurgery, requiring delicate motor skills. Microsurgical dexterity can be improved with regular laboratory practice along with progressive surgical experience. Despite the acquired skills, some voluntary and involuntary external factors affect surgical performance such as sleep deprivation, caffeine, and preoperative physical exercise [1, 2, 7].

Alcohol consumption has been reported to influence surgical performance up to the following day with sleep deprivation enhancing the negative effects [4, 5, 9]. The previous studies are mainly based on laparoscopic tasks, while data regarding performance in microsurgery are scarce [13]. Though alcohol consumption is banned from professional environments for obvious reasons, small portions of wine or beer may be served with lunch in some cultures. Sometimes the surgical expertise of an off-duty neurosurgeon may be required in an urgent operation even after consuming a small amount of alcohol.

This study aims to clarify the effect of blood alcohol levels on executing micro- and macro surgical tasks in a surgical laboratory setting. We hypothesize that alcohol consumption leads to immediate impaired performance.

## Methods and materials

### Participants

Five neurosurgeons and one neurosurgery resident, all males with microsurgical experience of 2–10 years, participated in this study between May and June 2022. All the participants were included as authors of this study. Ethical permission was not applicable.

### Alcohol consumption

All participants consumed zero, one, two, three, and four doses of alcohol (0.33 l of beer with 4.6% ethanol content, approximately 14 g of ethanol) on separate days. One task without prior alcohol consumption was performed by all study participants at the end of the data collection. After a delay of 60–157 min from finishing the last dose, they performed macro- and microsurgical tasks. The timely delay between consumption and surgical tasks was introduced so that each task was started under peak alcohol levels [11], tested with alcoholmeter (ACE Y Alkoholtester, ACE GmbH, Freilassing, Germany). Breathalyzer result was recorded at the start and the finish of a surgical task.

### Macro surgical task

**Fig. 1:**
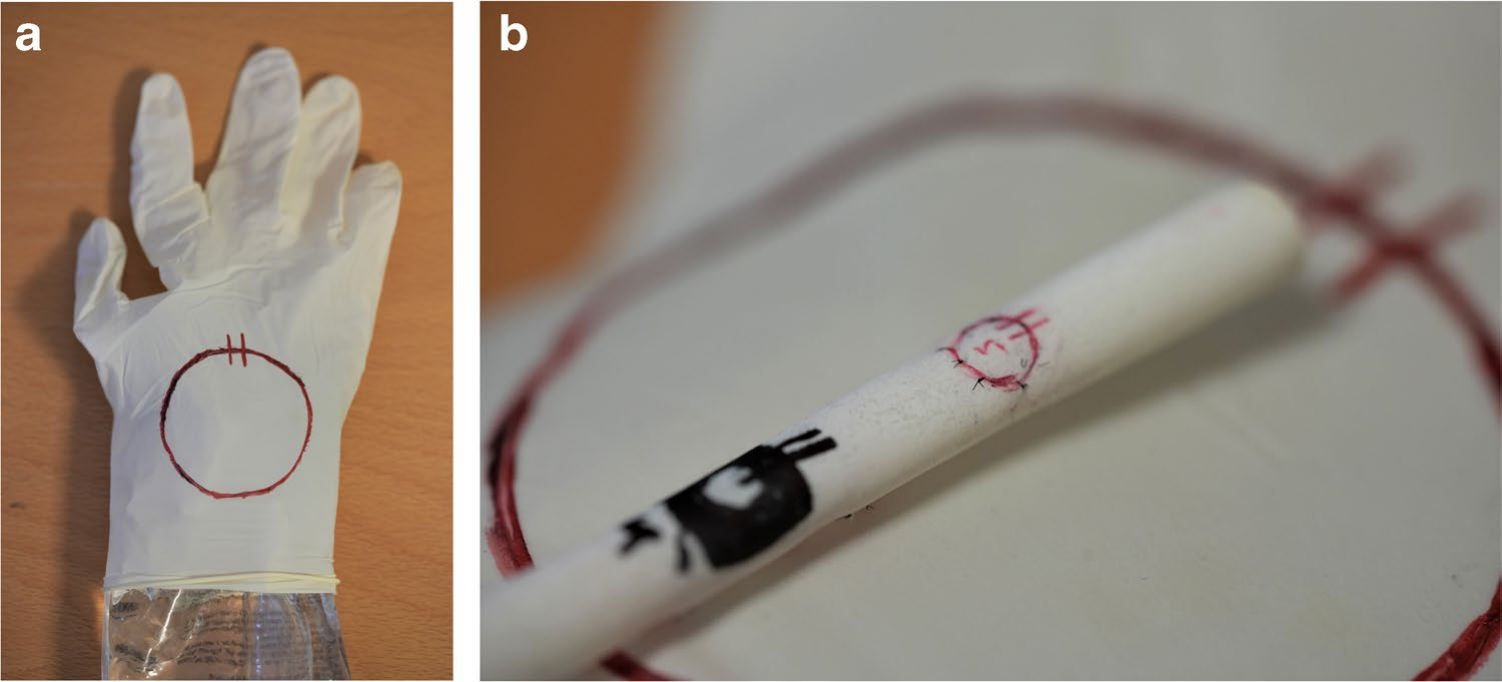
**a** and **b** The models used for macro- (**a**) and microsurgical (**b**) tasks

**Fig. 2:**
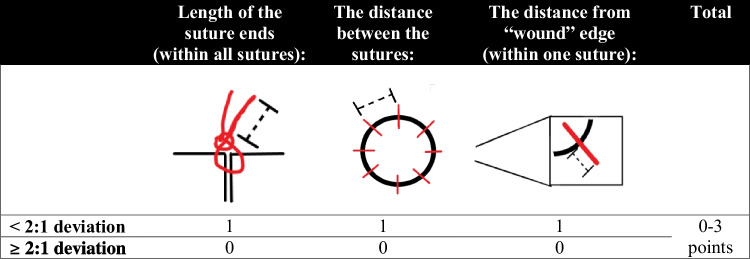
The instructions for calculation of quality points for each surgical task

The macrosurgical task was chosen to resemble suturing of dura mater. It was always performed prior to the microsurgical task. The task was to re-attach a circular piece (diameter 50 mm) cut from a latex surgical glow (Abena®, Aabenraa, Denmark) wrapped around a 1000-ml standard IV-infusion bag (Natriumklorid Baxter Viaflo®, Baxter, Deerfield, Illinois, USA) (Fig. 1). The re-attachment was performed with eight single interrupted 4–0 multifilament sutures (Vicryl Plus®, Ethicon, Johnson & Johnson, New Brunswick, New Jersey, USA). The duration from the first stitch to cutting the threads of the last stitch was measured. The quality of the sutures of each task was ranked between zero to three points according to Fig. 2. All the evaluations were done by a single author (C.E.). One point was lost if there were more than 2:1 deviations in the following parameters: (i) the length of the suture ends within all sutures (later referred as deviation of suture ends), (ii) the distance between any of the adjacent sutures (later referred as unequal distancing), (iii) the entry of suture in the opposite borders of simulated wound (later referred as deviation of suture entry).

### Microsurgical task

The microsurgical task was to re-attach a small circularly cut piece (diameter 4 mm) of a latex tube (Johnson & Johnson®, New Brunswick, New Jersey, USA; Fig. 1b). The re-attachment was performed with eight single interrupted monofilament 9–0 sutures (Dafilon®, B. Braun, Melsungen, Germany). The task was recorded with a surgical training microscope (M320, Leica Microsystems®, Wetzlar, Germany) and evaluated similarly to the macrosurgical task (Fig. 2). The frequency of physiological tremor was determined by selecting a representative segment of recording and then manually calculating the frequency (tremor/s). The amplitude of tremor was measured by comparing the movement of the tip of surgical forceps in reference to an immobile point on the latex tube.

### Statistical analysis

The distributions of time variables were plotted using histograms. Non-parametric time data are presented as medians with interquartile ranges [IQR], except when otherwise stated. Suturing quality points are reported as means with standard deviations (SD). The correlation between blood alcohol levels and time of surgical tasks were scatter plotted and assessed using Spearman’s correlation coefficient. Due to the relatively low number of observations, no *p*-values were calculated.

## Results

### Baseline data

This study included six participants with a mean age of 32.8 years (range 26–37) and a mean of 7.3 years (range 2–10) of neurosurgical training. A mean weekly consumption of 1.1 doses of alcohol (range 0–3) was reported by the participants during the last month prior to the experiment. A total of 47 and 46 macro- and microsurgical tasks were completed during the study, respectively.

### Macrosurgical suturing without previous alcohol consumption

Sixteen macrosurgical tasks were performed without prior alcohol consumption. The median duration was 7 min 39 s (IQR 318 to 535 s) with a mean loss of 0.20 quality points (SD 0.41) per task (Table 1). Two quality points were lost due to deviation of suture ends and one due to deviation of suture entry.

**Table 1:** The alcohol consumption in relation to the duration of microsurgical task and suture quality

	Number of alcohol doses (0.33 l, 4.6%)				
	0	1	2	3	4
Total number of accomplished tasks^a^	16	6	8	11	6
Blood alcohol prior macrosurgical task: median (IQR)	0	0.04‰ (0–0.09)	0.18‰ (0.10–0.23)	0.30‰ (0.27–0.42)	0.44‰ (0.39–0.48)
Time macrosurgical: median (IQR)	7 min 39 s (318–535 s)	7 min 14 s (390–530 s)	8 min 48 s (434–607 s)	5 min 56 s (318–468 s)	7 min 4 s (386–494 s)
Lost quality points—macrosurgical: mean (SD)	0.20/3.0 (0.41)	0.33/3.0 (0.52)	0.38/3.0 (0.52)	0.64/3.0 (0.67)	0.83/3.0 (0.75)
Blood alcohol prior microsurgical task: median (IQR)	0	0‰ (0–0.09)	0.15‰ (0–0.21)	0.32‰ (0.24–0.41)	0.44‰ (0.38–0.48)
Time microsurgical: median (IQR)	13 min 53 s (728–1249 s)	15 min 19 s (666–1121 s)	14 min 12 s (580–1178 s)	11 min 49 s (654–761 s)	15 min 12 s (742–1075 s)
Lost quality points—microsurgical: mean (SD)	0.67/3.0 (0.62)	0.50/3.0 (0.84)	0.63/3.0 (0.52)	0.70/3.0 (0.48)	0.33/3.0 (0.52)
Frequency of tremor in microsurgical task: median (IQR)^b^	5.0/s (4.1–6.0)	3.8/s (3.0–4.6)	4.0/s (3.0–5.0)	4.0/s (3.1–5.3)	3.5/s (3.0–4.0)
Amplitude of tremor in microsurgical task: median (IQR)^b^	0.26 cm (0.18–0.32)	0.19 cm (0.09–0.23)	0.17 cm (0.13–0.20)	0.19 cm (0.14–0.32)	0.11 cm (0.07–0.19)

^a^One microsurgical task after three beers was missing

^b^Information not available on four and five participants who consumed from zero and three doses of alcohol, respectively

*SD*, standard deviation; *IQR*, interquartile range

### Microsurgical suturing without previous alcohol consumption

In the microsurgical tasks without prior alcohol consumption (*n* = 16), the median duration was 13 min 53 s [IQR 728 to 1249 s] and the mean number of lost quality points per one task was 0.67 points (SD 0.62, Table 1). Seven quality points were lost due to the deviation of suture ends and three due to the unequal distancing between sutures. Information on the quality points of one macro- and microsurgical task performed sober were missing. The median duration for the six microsurgical tasks performed at the end of the data collection was 12 min 38 s [IQR 621 to 1086 s] and the mean number of lost quality points per task was 0.40 points (SD 0.55).

### Macrosurgical suturing following alcohol consumption

**Fig. 3:**
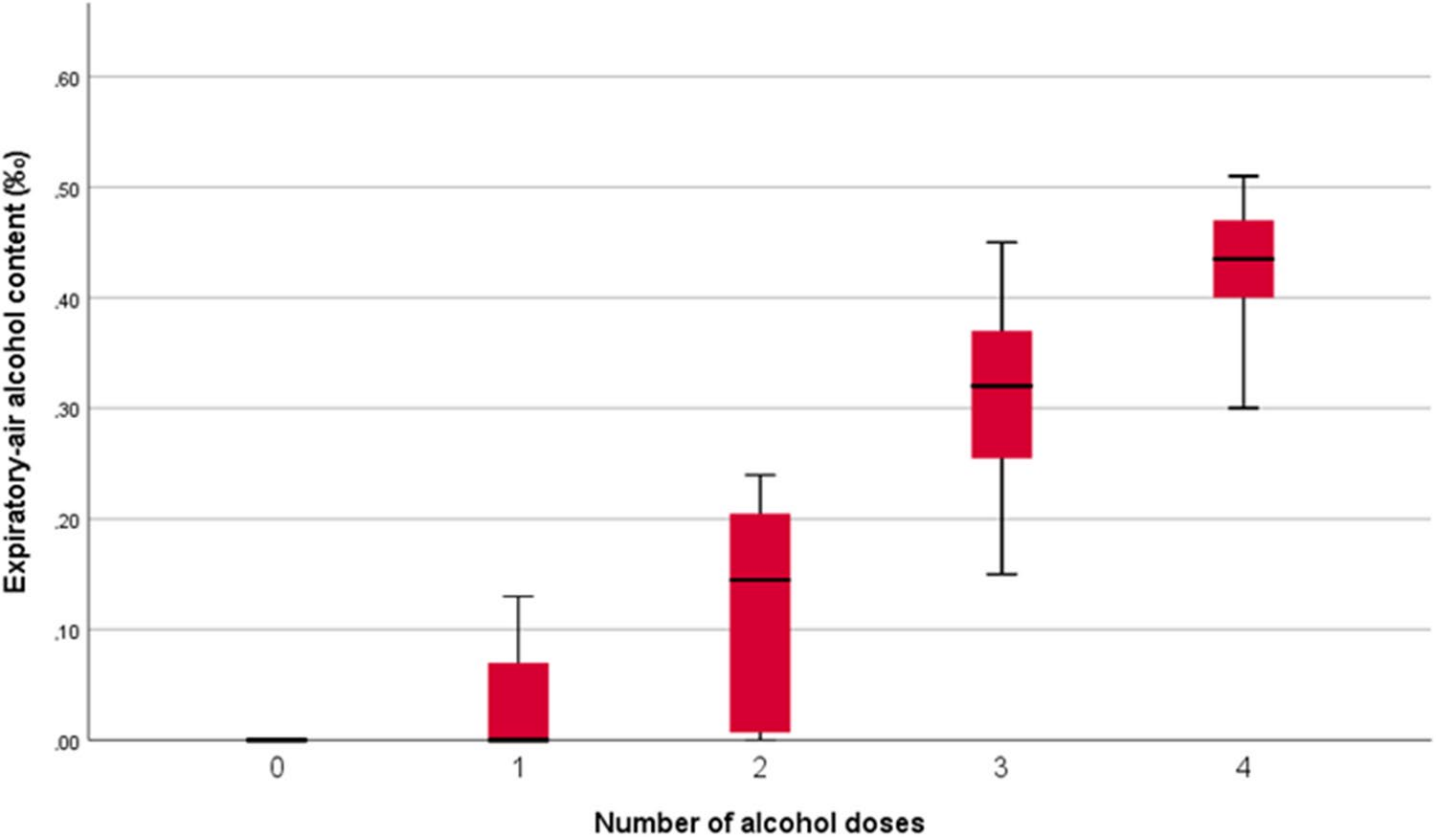
The relationship between blood alcohol level and the number of consumed alcohol doses

**Fig. 4:**
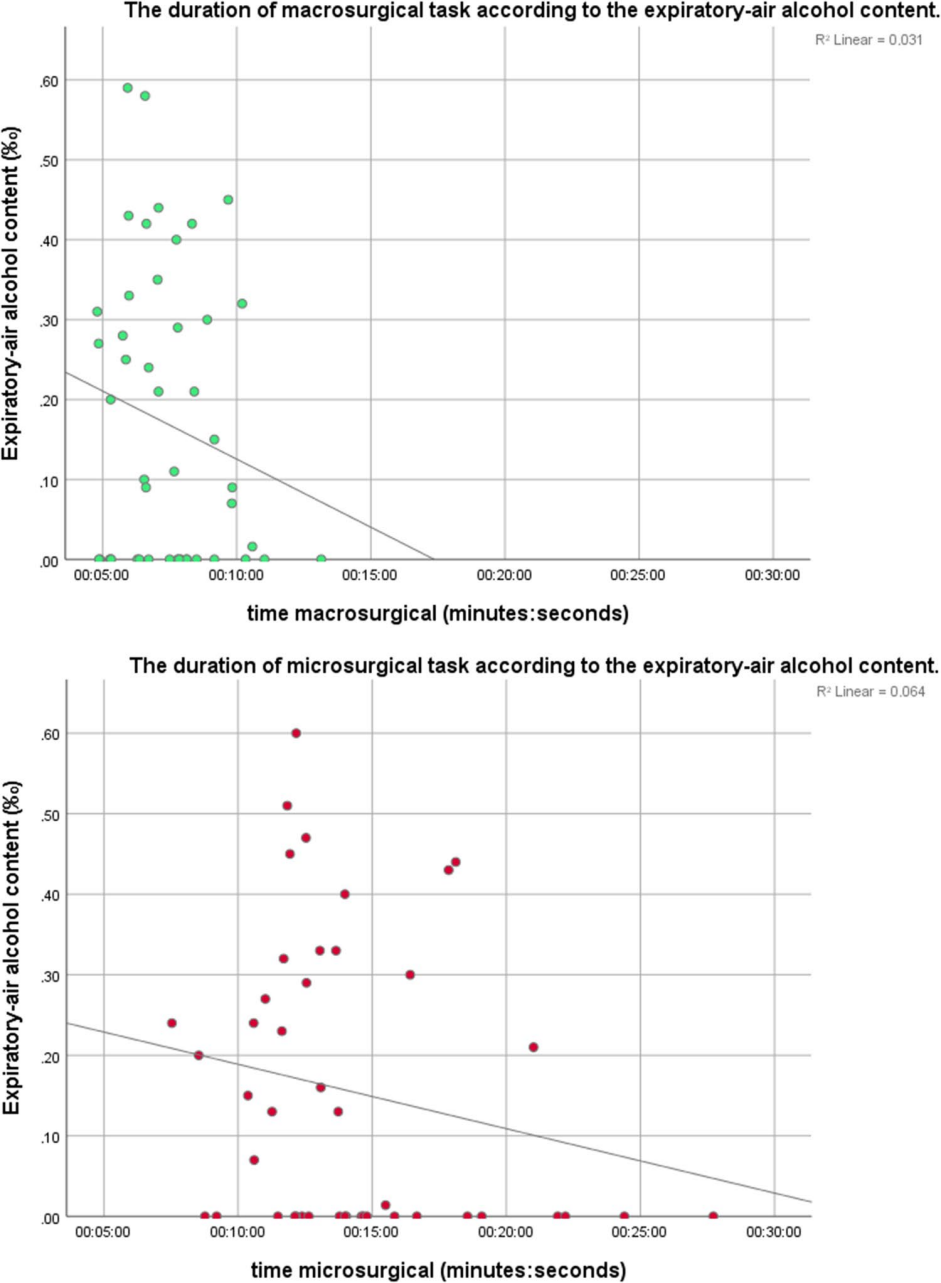
The relationship between blood alcohol level and duration of macro- and microsurgical task

A total of six, eight, eleven, and six macrosurgical tasks were performed after one to four doses of alcohol, respectively (Table 1). The mean duration from finishing the last dose of alcohol to the beginning of the macro surgical task was 84 min (range 60–157 min). The fastest times to complete the macrosurgical task (median 5 min 56 s, [IQR 318 to 468 s]) were seen after three doses of alcohol with median blood alcohol levels of 0.30‰ (Figs. 3 and 4). The slowest times (median 8 min 48 s, [IQR 434 to 607 s]) were observed after two doses of alcohol with median blood alcohol level of 0.18‰. The best quality of sutures was noted in tasks performed without alcohol consumption (mean 0.20 [SD 0.41] quality points lost per task) and the worst after four doses of alcohol (mean 0.83 [SD 0.75] quality points lost, median blood alcohol level 0.44‰). The most common reason for losing a quality point was deviation of suture ends reported after zero (2/15), one (2/6) and four (4/6) doses of alcohol, and unequal distancing after two (2/8) and three (4/11) doses of alcohol.

### Microsurgical suturing following alcohol consumption

Six, eight, ten, and six microsurgical tasks were completed after one to four doses of alcohol, respectively. The fastest (median 11 min 49 s, [IQR 654 to 761 s]) and the slowest (median 15 min 19 s, [IQR 666 to 1121 s]) performances were recorded after consuming three doses (median blood alcohol level 0.32‰) and one dose of alcohol (median blood alcohol level 0‰), respectively (Table 1, Figs. 3 and 4). The best quality of sutures was observed after consuming four doses of alcohol (mean 0.33 [SD 0.52] quality points lost per task, median blood alcohol level 0.44‰), whereas the worst quality was observed after three doses (mean 0.70 [SD 0.48] quality points lost). The most common reason for losing quality points was the deviation of suture ends after zero (7/15), three (6/10) and four (1/6) doses of alcohol, and unequal distancing of sutures after one (2/6) and two (3/8) doses of alcohol. The effect of alcohol consumption (doses 0, 1, 3, and 4) on the microsurgical performance of one study participant is demonstrated in the video 1.

### Analysis of physiological tremor

Analyses of the physiological tremor during the microsurgical task was attainable for twelve, six, eight, six, and six tasks following the consumption of zero to four doses of alcohol, respectively (Table 1). Four and five tasks following zero and three doses of alcohol, respectively, were not included to the analyses due to failure in video recording. The median frequency of tremor was the lowest after four doses of alcohol (3.5/s, [IQR 3.0 to 4.0]) and highest following zero doses (5.0/s, [IQR 4.1 to 6.0]). The amplitude of tremor was the lowest after four doses of alcohol (median 0.11 cm, [IQR 0.07 to 0.19]) and highest when performed sober (median 0.26 cm, [IQR 0.18 to 0.32]).

## Discussion

Contrary to our hypothesis, less than 0.50‰ blood alcohol level did not negatively affect the performance in microsurgical suturing. Instead, the suturing was faster with higher alcohol dosages in general. However, with the highest alcohol dosages, the quality of sutures deteriorated rather linearly in macrosurgical tasks but remained similar or were even better in microsurgical tasks. In neurosurgery, it is possible to encounter highly complex emergency situations of different subspecialities (e.g., vascular neurosurgery) where a specific off-duty surgeon with right skillset needs to be invited, possibly even after consuming some alcohol. In addition, in some cultures, it is generally acceptable to drink small doses of alcohol while dining during working hours. In this study, we aimed to find a threshold for unsafe microsurgical suturing under the influence of increasing alcohol levels. The highest amount of consumed alcohol was limited to four doses since it was anticipated that no one would even consider operating after more. Regardless of these results, we do not encourage anyone to consume alcohol prior to performing surgery and there is a zero tolerance for alcohol consumption during working hours in our department. Surgery does not consist of the sole manual act, but it takes planning, decision-making, anticipation, and emotional stability to safely conduct a surgical procedure. Even if alcohol consumption would improve microsurgical motor skills, the effects on planning the surgery and intraoperative decision-making can be detrimental.

The reason for the study hypothesis to fail may be explained by multiple factors. Firstly, the highest alcohol dosages were consumed at the end of the data collection, and thus, learning effect might have compensated the negative effects of alcohol. Even with increasing alcohol levels, both the time for suturing and the quality of the sutures improved after some repetitions of the microsurgical exercises. Second, being aware of consuming higher alcohol dosages, participants tended to concentrate more on the suturing as when operating under sleep deprivation. Third, it appeared that the higher alcohol dosages were associated with finding a “flow state” and not getting hung up on irrelevant details too much. Fourth, the tremor amplitude and even the frequency were generally lower after consuming one to four doses of alcohol, which could explain the relatively faster completion of macro- and microsurgical tasks in these cases. For easiness in use, we applied a standardized unit of alcohol consumption which might result in different blood alcohol levels depending on, e.g., participant’s body weight and speed of hepatic metabolization. Even after consumption of maximal dose (i.e., 4 small beers), blood alcohol content of all the participants decreased to less than 0.5‰ within 90 min, which in many countries is the limit for driving.

Opposing the findings of this study, some previous authors have reported that alcohol consumption may negatively affect one’s surgical performance within 1–2 h after consumption and up until the next day [1, 3, 8]. However, the effects on surgical dexterity and performance are not well described. Dorafshar et al. prospectively studied the effect of alcohol consumption on surgical performance in simulated laparoscopic procedures performed by 28 male medical students (mean age 22.4 years) in favor of the sober group (number of errors and excessive use of diathermy) [4]. The difference remained after adjustments for sleep deficits and experience level. Kocher et al. found similar trends in a smaller group of test subjects (5 surgeons, all men, aged 31–40 years) for which consuming higher doses of alcohol (mean 10.3 units and 0.86‰ expiratory-air alcohol content) reduced surgical performance [9]. When looking at microsurgical skill, Zyluk et al. prospectively studied the effect of 0.5 l of beer on ten medical students (20% women) suturing a latex glove [13]. No significant differences in time to complete the task were noted but the quality of sutures was not assessed. In some sports, e.g., sharpshooting, alcohol is considered to positively affect performance and is therefore banned as a doping substance.

It has been hypothesized that besides affecting concentration, alcohol may stimulate the physiological tremor [12]. However, supporting the findings of the present study, there are also data suggesting that alcohol in limited doses can even suppress physiologic tremor. In addition, suppression of the amplitude of essential tremor has been reported following ingesting of alcohol [6]. Lakie et al. reported that small alcohol doses (20–60 ml) decreased the amplitude of physiological tremor but did not change the frequency in a sample of 10 participants (60% women, mean age 22 years) 30–60 min after consumption [10]. The decrease in the tremor amplitude was the highest after 60 ml of alcohol but the effect was not linear [10].

### Limitations

This study has some limitations. Firstly, the study was performed in a laboratory setting, so the results cannot be directly extrapolatable to clinical practice. The results demonstrate the alcohol effects on the manual performance only, not the intellectual aspect of performing surgery. Meticulous preoperative planning as well as intraoperative decision-making and prevention of immediate complications were not evaluated in this study setting. Second, due to the limited number of participants (*n* = 6), the effect on surgical performance only applies in men younger than 40 years of age. More inclusive cohorts are required to learn about the effects of alcohol on microsurgical performance among surgeons with different age, sex, and physiological condition (e.g., delay from the last meal). Even then, the unique metabolism of blood alcohol between different individuals cannot be anticipated. Third, the repetition of the tasks may have affected the results since most of the tasks without prior alcohol consumption (10/16) were arranged at the beginning of the trial. However, each participant performed one sober microsurgical task at the end of the data collection. The suturing was faster and suture quality better during those tasks when compared to all tasks performed sober, which supports the existence of a learning curve. The fastest suturing times per participant were recorded after 82% of the macro surgical and 85% of the microsurgical tasks. In the optimal setting, the amount of consumed alcohol would have been randomized in relation to the phase of the data collection. Fourth, due to relatively short duration of microsurgical task (on average 10–15 min), the effect of alcohol on fatigue was not demonstrated in this study. Last, whether these results are convergent in other types of surgical tasks, e.g., endoscopic, exoscopic, or endovascular which require close observation of the digital monitors, cannot be confirmed. We hereby strongly discourage anyone to perform surgery on patients under the influence of alcohol.

### Strengths

Besides the limitations, the present study has strengths compared to the previous literature. The integration and comparison of both macro- and microsurgical tasks, of which the latter was performed with a surgical training microscope, enabled us to shed light on the effects of alcohol consumption in two rather different surgical skill sets. To the best of our knowledge, an operating microscope has been used in only one study before to assess alcohol’s effects on surgical performance. By evaluating the quality of work, we included an outcome that is probably more prone to the effects of alcohol compared to, e.g., speed of completing surgical tasks. Eventually, the analysis of physiological tremor enabled us to document a potential mechanism behind observed effects of alcohol on surgical performance.

## Conclusions

Consuming relatively small doses of alcohol, up to one liter of beer, did not clearly deteriorate the manual performance in microsurgical laboratory work. The reduction of physiological tremor may partially explain this finding. Information on other core components of performing a surgery, e.g. preoperative planning and intraoperative decision-making were not evaluated in this study.

## Supplementary Information

Below is the link to the electronic supplementary material.

Supplementary file1 (WMV 19357 KB)

## Abbreviations

IQR: Interquartile range
SD: Standard deviation
